# Coconut Allergy Revisited

**DOI:** 10.3390/children4100085

**Published:** 2017-09-29

**Authors:** Katherine Anagnostou

**Affiliations:** 1 Department of Pediatrics, Section of Immunology, Allergy and Rheumatology, Texas Children’s Hospital, Feigin Center, 1102 Bates Avenue, Suite 330, MS: BCM320, Houston, TX 77030, USA; Aikaterini.Anagnostou@bcm.edu; Tel.: +1-832-824-1319; 2 Department of Pediatrics, Section of Immunology, Allergy and Rheumatology, Baylor College of Medicine, Houston, TX 77030, USA

**Keywords:** food allergy, coconut allergy, anaphylaxis, allergic reaction, skin prick tests

## Abstract

Despite concerns voiced often by food-allergic patients, allergy to coconut is rare, not directly associated with nut allergy and few cases are reported so far in the literature. We present an interesting case of coconut allergy in a child that was previously tolerant to coconut and regularly exposed via both the skin and gastrointestinal route.

A common concern of food allergic patients (especially those allergic to nuts) is whether they can safely consume coconut products. Despite coconut allergy being rare, there is a misconception that if a patient is allergic to nuts, they are at high risk of experiencing allergic reactions to coconut; as a consequence, nut-allergic patients are not uncommonly advised to avoid coconut.

Coconut (*Cocos nucifera*) is a fruit (not a nut) that belongs to the Aracaceae (palms) plant family. The term is derived from the 16th-century Portuguese and Spanish word ‘coco’ meaning ‘head’ or ‘skull’. The oil and milk derived from coconut are commonly used in cooking and frying, as well as in soaps, cosmetics and other skin care products.

Reports of immunoglobulin E (IgE)-mediated coconut allergy are rare and only a handful of cases have been reported in the literature in adults and children [1,2,3,4]. However, despite the low prevalence of coconut allergy, reactions tend to be systemic and all cases reported so far have involved anaphylactic reactions. In the United States, coconut must be disclosed as an ingredient on package labels.

We report a case of coconut allergy in a school-age child, who presented with a history of recurrent allergic reactions to coconut. At the age of 6 years, the patient experienced generalized urticaria to coconut oil applied on the skin. Prior to this reaction, coconut oil had been applied multiple times a week on the skin, since the age of 2 weeks old, without any problems. The patient was also eating coconut regularly without allergic reactions. A second episode of skin reaction (widespread hives) was noted two weeks after the first one, again following application of coconut oil on the skin. Soon after these two episodes, the patient complained of a ‘scratchy throat’ after eating coconut. The parents also report one episode when the child ate a few spoonfuls of coconut ice-cream and, 20–30 min later, complained of throat itching and severe abdominal pain with subsequent vomiting and diarrhea. There were no respiratory symptoms on that occasion. The reaction was treated successfully with antihistamines. At the age of 6 and a half, the patient ate an oatmeal/raisin cookie that also contained coconut. Throat itching developed and was treated successfully with antihistamines. More recently however, while at school, the patient inadvertently ate another oatmeal/raisin cookie containing coconut and developed a scratchy throat. This was followed by vomiting and wheeze. Both bronchodilators and antihistamines were administered and the patient was taken to a medical facility. By the time of arrival, the wheeze had resolved and all other symptoms of anaphylaxis had also settled, so no further treatment was required.

In terms of atopic background, the patient did not report any other food allergies and had been eating peanuts, almonds, hazelnuts, cashews, pistachios, pecans and walnuts regularly without any problems or reactions. With regards to nasal allergies, parents reported mild, occasional, nasal congestion, not requiring treatment. No food pollen syndrome symptoms to any fresh fruits or raw vegetables were reported. The child had experienced allergic reactions to cats in the past with skin and upper respiratory/eye symptoms. With regards to respiratory symptoms, bronchodilators were prescribed at the age of 6 years, following an upper respiratory infection with persistent cough. These were not required on a regular basis. Mild infantile eczema was noted shortly after birth, mostly in the form of dry skin, for which coconut oil was applied. On examination in clinic, mild flexural eczema was noted on the antecubital fossae and popliteal fossae, well controlled with regular moisturizer.

Skin prick testing to coconut commercial extract showed a strongly positive result (20 mm wheal). Cat and dog skin test results were also positive (see Table 1 below).

Coconut allergens have previously been identified as Coc n2, a 7S globulin; and Coc n4, an 11S globulin [5,6]. Cross-reactivity between coconut and tree nuts/lentils has been described in the literature (due to the 7S and 11S globulins) [4,7,8], but a retrospective study from the US has previously reported that children with sensitization or allergy to peanuts or tree nuts are not more likely to be sensitized or allergic to coconut [9]. We note that the official allergen nomenclature subcommittee, the International Union of Immunological Societies (IUIS, http://www.allergen.org), currently includes only the 7S globulin allergen, also known as vicilin-like, and named Coc n1 for coconut.

What is interesting in our case is that coconut allergy developed in a child that was regularly exposed to coconut allergen previously without reaction. This involved skin exposure in the form of coconut oil since the age of two weeks and subsequently also via the oral route, tolerating coconut and coconut-containing products, until the age of 6 years. This pattern of coconut allergy development has not been reported previously in children, to our knowledge. It has been shown however, for peanut allergy, that sensitization may occur via the skin (without concurrent oral exposure), due to topical exposure from a very early age [10]. In our case, there was confirmed oral tolerance to coconut for some years prior to reactions occurring.

In summary, we report a case of coconut allergy, presenting in a 6-year-old child, despite previous regular exposure to coconut via both the cutaneous and oral route. Our knowledge in coconut allergy is limited as only a very small number of patients are affected. Given the increasing use of commercially available coconut products, it is important to be aware of the allergenic potential of coconut, even if the allergen has previously been tolerated.

## Figures and Tables

**Table 1:** Skin prick testing results. Measurements represent the mean of 2 diameters. Test results to other aeroallergens (including pollens and house dust mite) were negative (data not shown).

Allergen	Wheal/Flare (mm)
Control positive skin prick test	12/20
Control negative skin prick test	0/0
Coconut	20/26
Cat	15/20 P
Dog	6/8 P
Mold mix #1	4/4
Mold mix #2	3/3

P: Pseudopod formation.
